# REMITTENT IDIOPATHIC NECROTIZING ACROCYANOSIS - A RARE ENTITY

**DOI:** 10.4103/0019-5154.60362

**Published:** 2010

**Authors:** Sudip Das, Alok Kumar Roy, Arunasis Maiti

**Affiliations:** *From Department of Dermatology, NRS Medical College and Hospital, Kolkata, India.*

**Keywords:** *Delayed onset*, *male patient*, *diltiazem and pentoxiphylline*, *remittent idiopathic necrotizing acrocyanosis*

## Abstract

Remittent idiopathic necrotizing acrocyanosis is a very rare condition characterized by persistent systemic cyanotic or erythrocyanotic discoloration of hands and feet. It is associated with pain, tenderness of fingers and toes and may present as ulceration or gangrene of extremities. It is aggravated with cold exposure but persists even in summer. Acrocyanosis is not due to any systemic disease; peripheral arteriolar constriction with secondary vasodilatation due to disordered vascular tone of unknown etiology has been postulated. It responds to peripheral vasodilator drug but usually needs continuous long term therapy along with avoidance of cold exposure. We report the case of a 53-year-old male farmer with remittent necrotizing acrocyanosis.

## Introduction

Acrocyanosis is an uncommon condition characterized by symmetric coolness and violaceous discoloration of the hand and foot.[1] Affected skin is cyanotic or erythrocyanotic with mottled patterns. It is a persistent disorder without episodic triphasic color response. Acrocyanosis is usually painless. Trophic changes and ulceration are extremely rare except in remittent necrotizing variety. It mainly affects the hands; sometimes, feet and face may be involved. Acrocyanosis occurs due to decrease in the amount of oxygen delivered to the extremities. The exact etiology is unknown. Peripheral arterial vasoconstriction due to increased tone of the arterioles associated with secondary vasodilatation of capillaries and subpapillary venous plexus has been postulated.[2,3] The cause of the disordered vascular tone is unknown. Changes of blood viscosity have been reported.

The affected areas become cold and sweaty; localized swelling may also occur. Emotion and cold temperature can worsen the symptoms while warmth can decrease symptoms. The changes may be transient after cold exposure but frequently persist during winter and even throughout the summer months.[4] The disease is seen mainly in women and often a family history of the disorder is present, indicating a genetic basis. This disorder usually starts in adolescence (less than 30 years old) and persists into adult life,[4] often, improving with age.[2]

The acrocyanosis patient's pulse is normal and there is no venous occlusion which rules out obstructive disease. Acrocyanosis may be idiopathic or secondary due to connective tissue disease, cryoglobulinemia, paraproteinaemia, essential thrombocytopenia, anorexia nervosa, drugs (butyl nitrate, interferon 2 alpha etc), neurological and psychiatric conditions. In idiopathic acrocyanosis, patient is otherwise asymptomatic without any specific laboratory changes.

There is no causative medical or surgical treatment for acrocyanosis. Lifestyle change, avoidance of cold temperature, protective clothing often improves the discoloration. Calcium channel blockers like nifedipine and diltiazem are not very effective as with other episodic peripheral vascular diseases. Topically applied nicotinic acid derivatives and minoxidil can be beneficial.[4] Sympathectomy or disrupting the fibers of the sympathetic nervous system to the area will usually alleviate the cyanosis but such an extreme procedure would rarely be appropriate.[2] Although there is no cure, the prognosis is otherwise normal without increased risk of death and complications.

## Case Report

A 53-year-old male farmer presented at our OPD with bluish discoloration of fingers of both hands since two years. Feet and face were not involved. It started with mild bluish discoloration of fingertips, which was associated with mild swelling of finger and hand. Discoloration persisted without remission even in summer. There was no history of any episodic appearance of symptoms or triphagic color change (blanching, pallor followed by bluish discolouration and pink after warming). Patient had no other systemic complaint. There was no history of smoking, alcoholism, drug intake or other systemic disease. There was no family history of such a disease.

**Figure 1:**
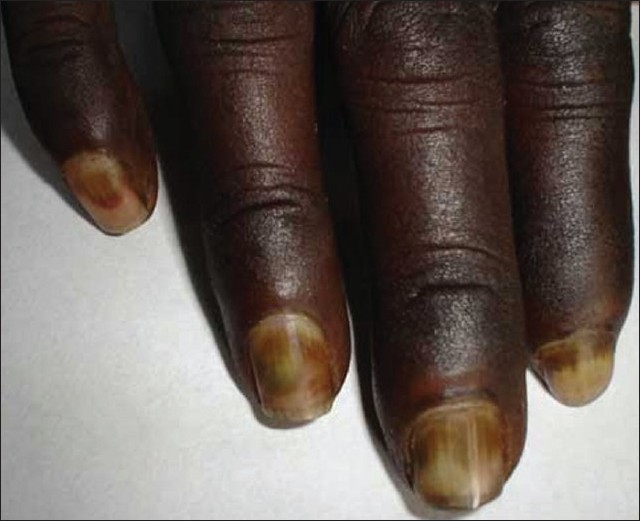
Acrocyanosis

**Figure 2:**
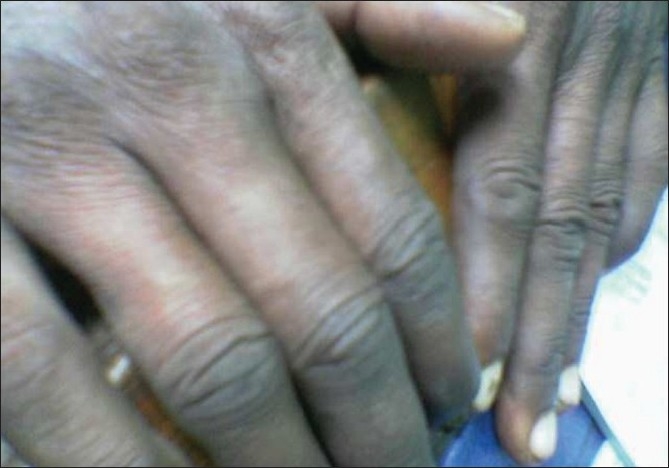
Post treatment acrocyanosis

**Figure 3:**
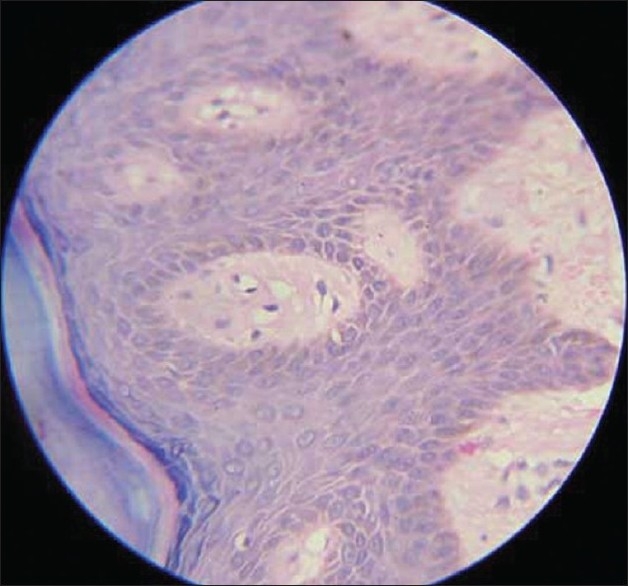
Histopathology of acrocyanosis

On examination, violaceous discoloration seen over both hands, but the right hand was more severely affected. There was pulp atrophy and mild ulceration of two fingers (index and middle) of the right hand [Figures 1 and 2]. Tips of the fingers were painful and tender and the whole hand was cold but without much sweating. Sclerodactly like skin stiffening was not felt over fingers. Arterial pulses were normal on both sides. Adson's test, Tinel's test and Phalen's test were negative. Laboratory investigation showed only mild anemia (11 gm %) ANF, RA factor, antiphospholipid antibody, cryoglobulin were negative. Doppler study was normal. Biopsy showed mild perivascular infiltration, a few thrombotic vessels and mild dermal edema [Figure 3]. X-ray cervical spine was normal.

## Discussion

Idiopathic acrocyanosis is a very rare disease. We present a case of persistent symmetric violaceous discoloration of hand without any episodic triphasic change indicating acrocyanosis. We did not find any systemic regional abnormality clinically and laboratory investigation was not significant. The case was diagnosed as remittent necrotizing acrocyanosis since discoloration was associated with pain, tenderness, ulceration, usually not present in most of the acrocyanosis patients. We counseled the patient and advised him to avoid cold and use cotton gloves. He was treated with diltiazem 60 mg twice daily and pentoxiphylline 400 mg thrice daily for six weeks. There was moderate improvement in pain and lesion became stationary with nonprogression of ulceration. But when the dose of diltiazem was reduced and pentoxiphylline stopped, pain on finger recurred. This compelled us to increase the dose of the drug and restart pentoxiphylline.

## Conclusion

We report this case as remittent necrotizing acrocyanosis, which is very rare. Acrocyanosis is less common in males and initiation is in older age. Response to treatment is satisfying with the present regime but he needs further follow-up.

## References

[1] Nousari HC, Kimyai-Asadi A, Anhalt GJ (2001). Chronic idiopathic Acrocyanosis. J Am Acad Dermatol.

[2] Creager MA, Dzau VJ, Kasper DL, Fauci AS, Lango DL, Braunwald E, Hauser SL, Jameson JL (2005). “Vascular Disease of the Extremity”. Harrison's Principles of Internal Medicine.

[3] Terence RJ, Freedberg IM Skin changes due to mechanical and physical factors. Fitzpatrick's Dermatology in general medicine.

[4] Dowd PM, Burns T Reaction to cold. Rook's Text book of Dermatology.

